# Mohs micrographic surgery versus wide local excision for the treatment of atypical fibroxanthoma: A retrospective cohort analysis

**DOI:** 10.1016/j.jdin.2023.06.003

**Published:** 2023-06-15

**Authors:** Summer N. Meyer, Yunyi Ren, Sandra Taylor, Maija Kiuru, Daniel B. Eisen

**Affiliations:** a Department of Dermatology, University of California, Davis School of Medicine, Sacramento, California; b Department of Public Health Sciences, University of California, Davis School of Medicine, Sacramento, California; c Department of Pathology and Laboratory Medicine, University of California, Davis School of Medicine, Sacramento, California

**Keywords:** atypical fibroxanthoma, dermatology surgery, Mohs micrographic surgery, pleomorphic dermal sarcoma, wide local excision

*To the Editor:* Atypical fibroxanthoma (AFX) is a rare pleomorphic, spindle cell neoplasm that classically presents as a solitary pink/red papule on the head or neck in elderly individuals.^1^ Current treatment guidelines recommend Mohs micrographic surgery (MMS) or wide local excision (WLE), yet MMS is generally preferred in clinical practice based on the limited data supporting superior recurrence rates. However, very few retrospective studies have compared these 2 surgical modalities, and some did not find meaningful differences in the rates of recurrence.^2^ The aim of this study was to compare the rates of recurrence and survival between MMS and WLE for AFX.

We retrospectively analyzed the surgical outcomes of 56 patients that underwent MMS (*n* = 50) or WLE (*n* = 6) for AFX removal at the University of California Davis Medical Center from 2008 to 2022.

Our results found lower recurrence rates for MMS (5.4%) compared to WLE (33.3%), albeit not significantly (*P* = .084, Table I). All recurrences occurred within the first 2 years of treatment (mean = 1.6 years). MMS was associated with better survival to recurrence (*P* = .024; Fig 1), yet no differences in the survival to death between WLE and MMS was observed (*P* = .87). Those with WLE had significantly larger tumors (26.0 vs 10.0 mm, *P* = .002), younger ages of diagnoses (61.0 vs 75.3 years, *P* = .015), and a larger proportion with prior radiation exposure to the AFX site (50.0% vs 4.0%, *P* = .007). Additionally, our univariate analysis, available in the supplemental materials, showed that higher Charlson Comorbidity Index scores were associated with recurrence (*P* = .015; Supplementary Table I footnote c, available via Mendeley at https://doi.org/10.17632/dksz5tw754.1).

**Table I:** Patient demographics and atypical fibroxanthoma characteristics by treatment type

Characteristics, *N* (%)	MMS (*N* = 50)	WLE (*N* = 6)∗	Total (*N* = 56)	*P* value
Age at diagnosis, y†	75.3 (12.5)	61.0 (18.1)	73.7 (13.7)	.015
Sex				.289
Male	42 (84.0)	4 (66.7)	46 (82.1)	
Female	8 (16.0)	2 (33.3)	10 (17.9)	
Race				N/A
White	45 (100.0)	6 (100.0)	51 (100.0)	
Not reported	5	0	5	
CCI score†^,^‡	7.4 (2.7)	6.7 (3.8)	7.3 (2.8)	.562
Prior skin cancer diagnosis	41 (82.0)	4 (66.7)	45 (80.4)	.586
SCC	31 (62.0)	4 (66.7)	35 (62.5)	
BCC	33 (66.0)	3 (50.0)	36 (64.3)	
Melanoma	10 (20.0)	2 (33.3)	12 (21.4)	
Tumor location				.008
Face	21 (42.0)	1 (16.7)	22 (39.3)	
Scalp	27 (54.0)	2 (33.3)	29 (51.8)	
Neck	0 (0)	1 (16.7)	1 (1.8)	
Trunk	0 (0)	1 (16.7)	1 (1.8)	
Upper extremity	1 (2.0)	1 (16.7)	2 (3.6)	
Lower extremity	1 (2.0)	0 (0)	1 (1.8)	
Radiation exposure to AFX site	2 (4.0)	3 (50.0)	5 (8.9)	.007
Immunosuppressed	3 (6.0)	1 (16.7)	4 (7.1)	.373
Tumor size at diagnosis, cm†	1.0 (0.8)	2.6 (2.6)	1.2 (1.1)	.002
Time to treatment, mo†	40.1 (35.8)	57.5 (55.9)	42.0 (38.2)	.295
Mohs stages	1.4 (0.5)	N/A	1.4 (0.5)	N/A
WLE margins, cm§	N/A	1.3 (0.5-2)	1.3 (0.5-2)	N/A
Up-diagnosis	3 (6.0)	1 (16.7)	4 (7.1)	.373
Up-diagnosis type				1.00
UPS	1 (2.0)	1 (16.7)	2 (3.6)	
MFH	1 (2.0)	0 (0.0)	1 (1.8)	
PDS	1 (2.0)	0 (0.0)	1 (1.8)	
Treatment sequela				.084
Distant recurrence/metastasis	1 (2.0)	0 (0.0)	1 (1.8)	
Local recurrence	2 (4.0)	2 (33.3)	4 (7.1)	
No return	47 (94.0)	4 (66.7)	51 (91.1)	
Time to recurrence, mo†	13.7 (6.5)	27 (29.7)	19 (17.2)	.322
No. of recurrences per patient†	2.7 (1.2)	1.0 (0.0)	2.0 (1.2)	.148
Follow-up status				1.00
Alive	42 (84.0)	5 (83.3)	47 (83.9)	
Dead due to other cause	8 (16.0)	1 (16.7)	9 (16.1)	
Follow up time, y†	4.4 (3.6)	3.3 (2.7)	4.3 (3.5)	.387

No significant differences were observed in the recurrence rates between groups. Those treated with WLE had significantly larger sized tumors (*P* = .002), younger ages of diagnosis (*P* = .015), and a larger proportion of patients that had prior radiation exposure to AFX site (*P* = .007). Notably, 96.0% of those with MMS had tumors located on the head and neck, compared to 66.7% in those with WLE. Two sample *t* tests were used to compare continuous variables, and Fisher’s exact tests were used to compare categorial variables between groups.

*AFX*, Atypical fibroxanthoma; *BCC*, basal cell carcinoma; *CCI*, Charlson Comorbidity Index; *MFH*, malignant fibrous histiocytoma; *MMS*, mohs micrographic surgery; *PDS*, pleomorphic dermal sarcoma; *SCC*, squamous cell carcinoma; *UPS*, undifferentiated pleomorphic sarcoma; *WLE*, wide local excision.

∗ Of the 6 WLE cases, 3 were performed by otolaryngology, and 1 by general surgery; 2 did not have this information available. WLE was recommended over Mohs in the 6 included cases for the following reasons: surrounding SCC and recurrent BCC (previously excised with postoperative radiotherapy) found on initial AFX biopsy (*n* = 1); rapidly enlarging and concern for depth of tumor (*n* = 2); concern for depth of tumor as AFX was transected at all margins on initial biopsy (*n* = 1); reason not reported (*n* = 2).

† Mean (standard deviation).

‡ CCI, or Charlson Comorbidity Index, represents the number of comorbid diseases in a patient and predicts the 10-year mortality. Higher scores represent a greater number and/or more severe comorbid diseases.

§ Mean (range).

**Fig 1:**
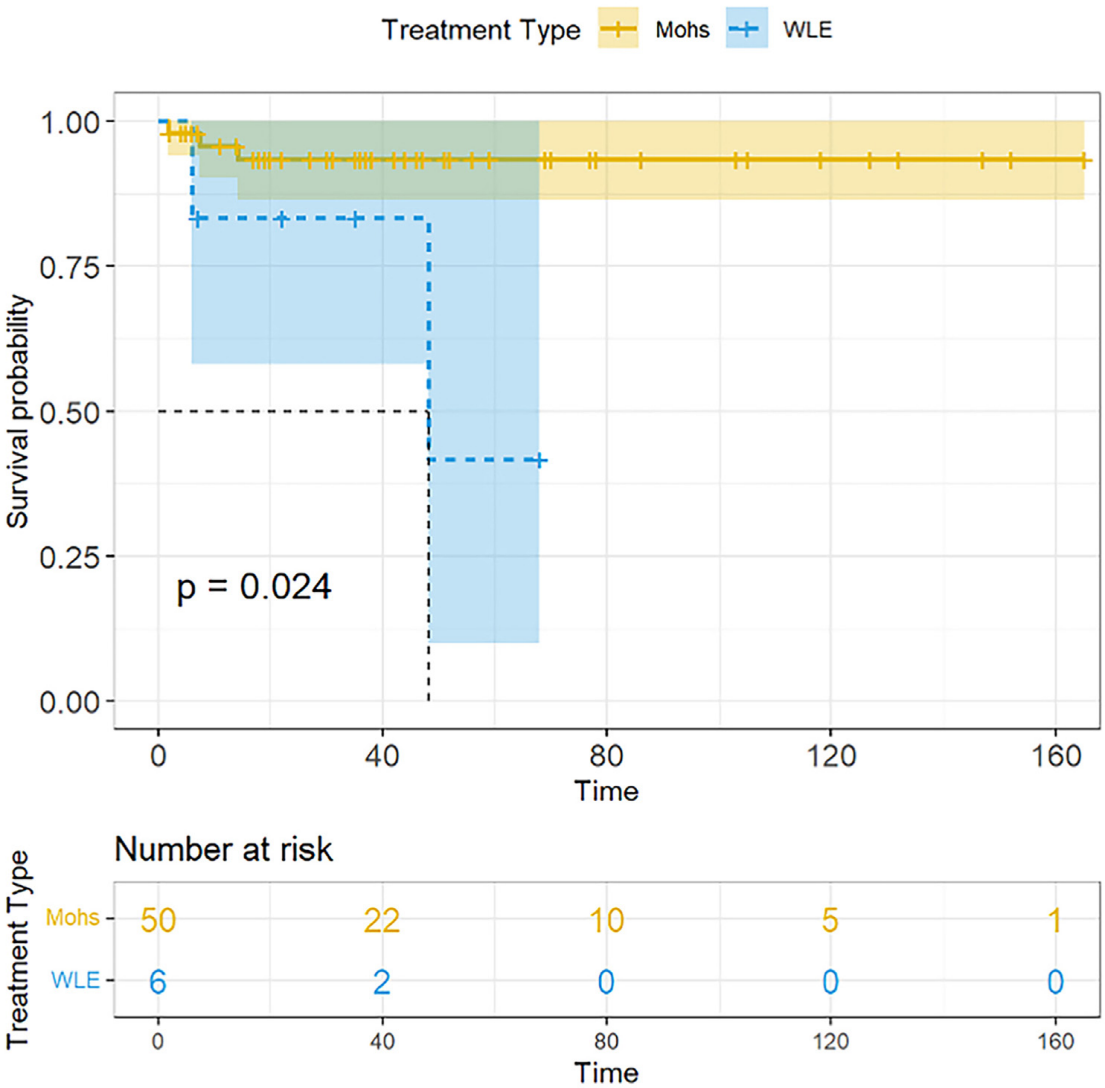
Atypical fibroxanthoma survival by treatment type. Kaplan-Meier estimate of survival to recurrence of patients with atypical fibroxanthoma treated with Mohs micrographic surgery (MMS) or wide local excision (WLE), with log-rank test *P* value. Not included is the Kaplan-Meier estimate of survival to death of patients with atypical fibroxanthoma treated with MMS vs WLE. Those treated with MMS had better recurrence free survival than did the WLE group (*P* = .024); no differences were observed in the survival to death between WLE and MMS (*P* = .87). The *blue* (WLE) and *yellow* (MMS) shaded regions represent the 95% confidence bands for the survival function. *WLE*, Wide local excision.

Our results did not find significant differences in the recurrence rates between MMS and WLE for AFX, which may be attributed to the small sample size of the WLE group. However, previous studies have demonstrated improved outcomes with MMS for AFX. A systematic review of 907 patients with AFX found significantly lower recurrence rates with MMS than with WLE (2.0% vs 8.7%).^3^ Another retrospective single center study similarly reported a 0% recurrence rate with MMS (*n* = 59) compared to 8.7% with WLE (*n* = 23).^3^ Alternatively, it is possible that larger and potentially more aggressive tumors are referred to WLE, contributing to these observed differences.

All recurrences occurred within 2 years after removal in our study, which is consistent with the literature that reports a <1% risk of recurrence after this time point^4^ and suggests that a follow-up period of at least 2 years should be considered. MMS was associated with better survival to recurrence than with WLE, which may reflect the smaller, and perhaps less invasive tumors in the MMS group. Furthermore, our univariate analysis showed that higher comorbidity scores were associated with recurrence, while prior reports suggest that ages >74 and male sex are also risk factors for recurrence.^2^

Limitations of this study included small sample sizes and a retrospective single center study design. Although MMS may offer improved recurrence rates for AFX, additional prospective randomized studies with larger sample sizes are warranted to make definitive conclusions.

## Conflicts of interest

None disclosed.
